# Supplementary material for: Optimizing Nitrogen Application for Jojoba under Intensive Cultivation
Source: Plants (Basel). 2023 Aug 31;12(17):3132. doi: 10.3390/plants12173132 (PMC10489925; doi:10.3390/plants12173132)
Supplement: Supplementary file 1 [file plants-12-03132-s001.zip › plants-2571162-supplementary.pdf]

Table S1. Effect of N fertilization rate on old (O) and young (Y) leaf mineral composition. NS, not significant; * significant (p < 0.05, Tukey- HSD); /, mineral concentration not measured. Different letters indicate significant differences (p < 0.05, Tukey-HSD) between treatments.

Treatment	Ca	Mg	Na	Cl	S	B	Cu	Fe	Mn	Zn	Mo
	%					mg/kg (ppm)					
**2016 O**											
N50	0.73	0.88	0.06	1.82	/	81.43	4.34	69.76	68.46	/	/
N150	0.79	0.89	0.06	1.87	/	79.32	4.00	60.53	57.98	/	/
N250	0.79	0.84	0.06	1.75	/	81.77	4.10	72.37	53.10	/	/
N370	0.77	0.86	0.06	1.84	/	76.86	3.74	67.65	74.29	/	/
N500	0.72	0.88	0.07	1.74	/	83.25	3.99	66.95	62.56	/	/
p<0.05	NS	NS	NS	NS		NS	NS	NS	NS		
**2018 O**											
N50	0.98	0.90	0.12	1.57	0.20	570.44	7.24	117.20	94.62 A	11.39	/
N150	0.93	0.84	0.09	1.58	0.21	507.45	6.43	117.30	73.91 AB	9.67	/
N250	0.96	0.84	0.11	1.51	0.22	486.17	8.00	122.04	83.08 AB	11.07	/
N370	1.11	0.79	0.15	1.41	0.23	508.77	6.74	116.89	57.32 B	10.12	/
N500	0.93	0.78	0.10	1.44	0.23	577.00	6.46	105.28	76.34 AB	9.60	/
p<0.05	NS	NS	NS	NS	NS	NS	NS	NS	*	NS	
**2018 Y**											
N50	0.68 C	0.80	0.05	1.87 A	0.19	332.25	4.06 A	40.61	93.71 A	17.47	/
N150	0.79 BC	0.79	0.04	1.75 AB	0.19	429.95	3.24 AB	42.47	75.72 AB	13.82	/
N250	0.80 B	0.81	0.04	1.83 AB	0.20	475.58	3.92 AB	38.26	87.88 AB	15.96	/
N370	0.94 A	0.74	0.05	1.59 B	0.20	490.60	3.47 AB	36.89	56.94 B	15.91	/
N500	0.84 AB	0.76	0.04	1.74 AB	0.20	429.18	3.04 B	37.57	78.38 AB	13.91	/
p<0.05	*	NS	NS	*	NS	NS	*	NS	*	NS	
**2020 O**											
N50	1.12	1.00	0.19	1.57	0.17 B	89.30	7.54 A	91.47	116.11	11.94	2.24 A
N150	1.12	0.92	0.18	1.57	0.21 A	82.41	6.95 A	81.59	85.60	12.58	1.86 AB
N250	1.29	1.01	0.18	1.45	0.19 AB	90.76	6.91 A	95.11	106.79	11.92	1.47 BC
N370	1.38	0.98	0.19	1.53	0.2 AB	81.45	5.29 B	72.77	95.10	10.45	1.16 C
N500	1.33	0.98	0.15	1.60	0.22 A	80.82	4.87 B	75.68	106.55	13.06	1.26 C
p<0.05	NS	NS	NS	NS	*	NS	*	NS	NS	NS	*
**2020 Y**											
N50	0.79 B	0.75	0.07 A	1.91	0.21 B	50.73	4.79 A	36.14	54.89 B	21.89	1.93 A
N150	0.89 AB	0.81	0.05 B	1.99	0.26 A	41.45	3.56 B	37.92	50.17 B	22.91	1.71 AB
N250	0.92 AB	0.83	0.04 B	2.13	0.22 AB	44.25	3.79 B	36.54	73.8 AB	20.91	1.41 BC
N370	1.02 A	0.84	0.04 B	2.00	0.22 AB	42.70	3.06 BC	32.06	99.8 A	19.65	1.14 C
N500	0.95 AB	0.81	0.04 B	2.25	0.23 AB	39.27	2.62 C	33.60	76.27 AB	19.58	1.27 C
p<0.05	*	NS	*	NS	*	NS	*	NS	*	NS	*
